# Parenchymal renal rupture due to an obstructive ureteric calculus in an incompletely duplicated renal pelvis and ureter

**DOI:** 10.1002/iju5.12697

**Published:** 2024-01-30

**Authors:** Moe Toyoshima, Daiki Ikarashi, Kie Sekiguchi, Tatsuya Kawamura, Arisa Machida, Takeshi Yamaguchi, Yumeka Arakawa, Akito Ito, Shigekatsu Maekawa, Wataru Obara

**Affiliations:** ^1^ Department of Urology Iwate Medical University School of Medicine Iwate Japan; ^2^ Department of Urology Iwate Prefectural Chubu Hospital Iwate Japan

**Keywords:** incompletely duplicated renal pelvis and ureter, parenchymal renal rupture, ureteral stent, ureteric calculus

## Abstract

**Introduction:**

Parenchymal renal rupture due to a ureteric calculus is extremely rare and an emergency.

**Case presentation:**

A 54‐year‐old man was brought to the emergency room with left back pain without trauma. Computed tomography showed left parenchymal renal rupture with an incompletely duplicated renal pelvis, ureter, and an 11‐mm ureteric calculus in the ureterovesical junction. A ureteral stent was placed, and the patient was treated conservatively as his vital signs were stable. We performed transurethral lithotripsy after resolution of the perirenal hematoma.

**Conclusion:**

To best of our knowledge, this report is the first to present a case of parenchymal renal rupture due to a ureteric calculus in an incompletely duplicated renal pelvis and ureter. Ureteric calculus within an incompletely duplicated renal pelvis and ureter is at risk of parenchymal renal rupture. Therefore, the aggressive treatment of ureteric calculus could be important.

**Abbreviation & Acronym**
CT: computed tomography

**Keynote message**

We demonstrated a case of parenchymal renal rupture due to a ureteric calculus in an incompletely duplicated renal pelvis and ureter. In systemic conditions where conservative treatment is possible, ureteral stenting for renal preservation is worthwhile.

## Introduction

Spontaneous parenchymal renal rupture is rare. Its incidence is low compared with renal pelvis rupture,^1^ and benign and malignant tumors are common causes of this condition. Four parenchymal renal rupture cases due to a ureteric calculus have been published.^2^, ^3^, ^4^, ^5^, ^6^ However, to the best of our knowledge, there have been no reports of parenchymal renal rupture associated with ureteric calculus in a duplicated renal pelvis and ureter.

The duplex kidney has two pyelocaliceal systems associated with a single ureter, partial ureteral duplication, or complete ureteral duplication.^7^, ^8^ Renal and ureteral duplication has been reported in 0.9% of autopsies with an incidence of 0.7%–4% and a higher prevalence in women and men.^9^ Ureteral duplication is usually asymptomatic and diagnosed incidentally; however, it can be associated with ureteric calculi, urinary tract infections, vesicoureteral reflux, ureteral varices, and other congenital complications.^10^ Herein, we report a parenchymal renal rupture due to an obstructive ureteric calculus in an incompletely duplicated renal pelvis and ureter.

## Case report

**Fig. 1:**
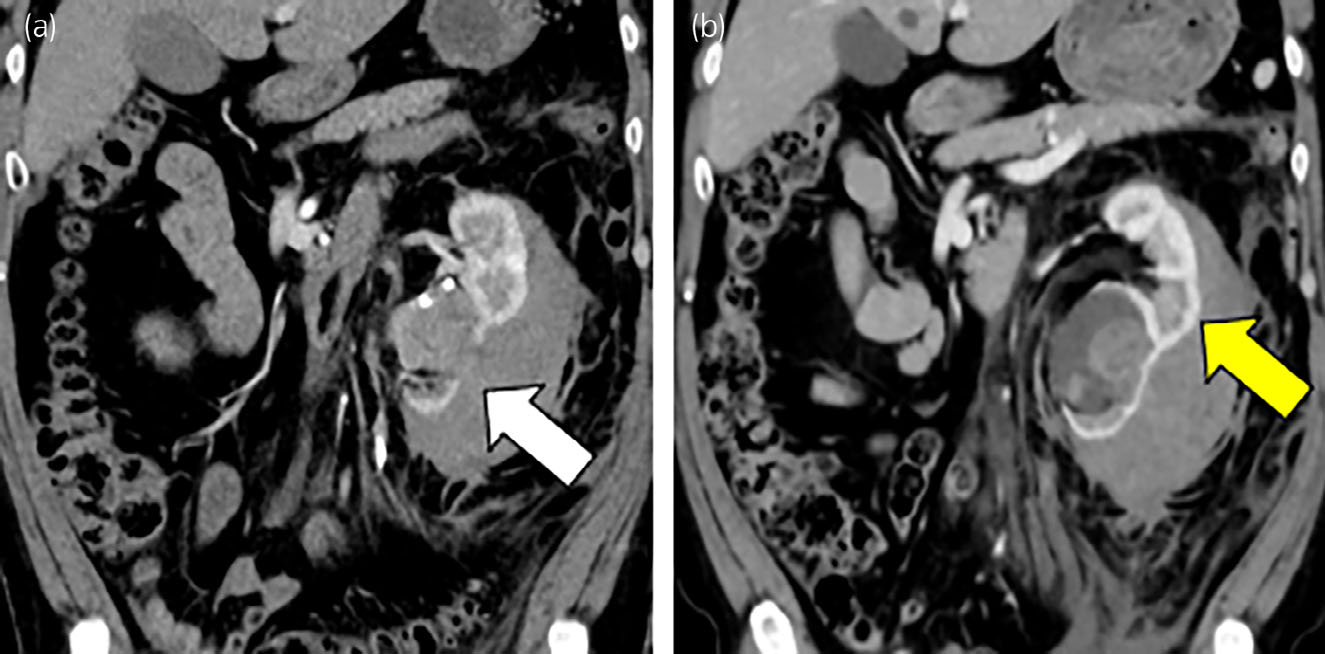
(a) Abdominal and pelvic enhanced CT in coronal view showing a rupture of the lower half of the left renal parenchyma with perirenal hematoma (white arrow). (b) The upper left half of the kidney is normal (yellow arrow).

**Fig. 2:**
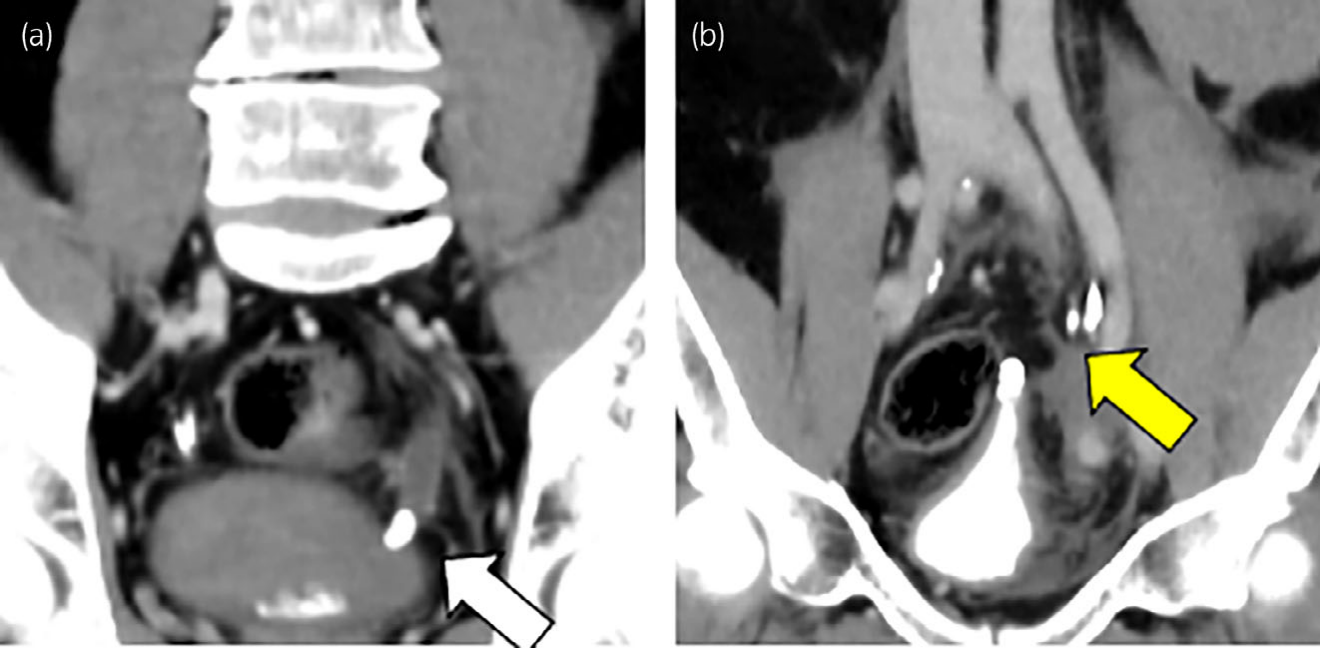
(a) Ureteric calculus of ~11 mm in the vesicoureteral transition area (white arrow). (b) Enhanced CT with excretory phase showing incompletely duplicated ureter (yellow arrow).

**Fig. 3:**
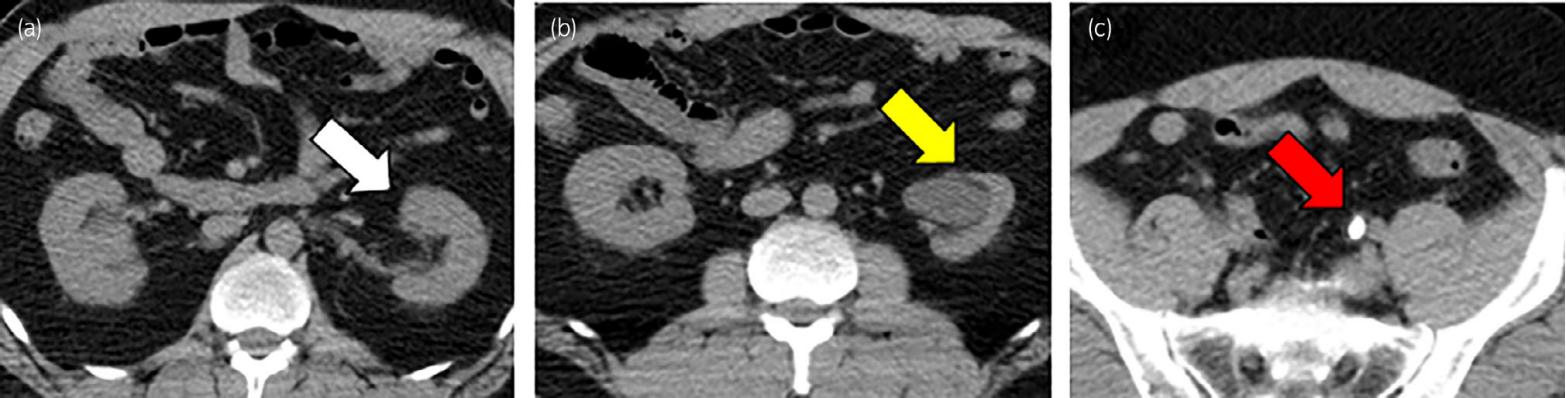
A follow‐up CT of a parotid carcinoma taken 10 months before the renal rupture showed (a) the upper half of the kidney retaining its renal cortex morphology (white arrow), while (b) the lower half of the kidney was atrophied (yellow arrow). (c) Ureteric calculus were identified upstream from the confluence of the lower half of the renal ureter (red arrow).

A 54‐year‐old man was brought to the emergency department with sudden left back pain and macrohematuria without the occasion of trauma. His medical history included hypertension, a parotid carcinoma, and renal calculi. Abdominal and pelvic enhanced CT showed a left parenchymal renal rupture and perirenal hematoma with a duplicated renal pelvis (Fig. 1). The size of perirenal hematoma was about a width of 55 mm, a height of 150 mm, and a depth of 105 mm. In addition, there was no hydronephrosis in the upper half of the left kidney. A ureteric calculus of 11 mm was found at the vesicoureteral junction with an incompletely duplicated ureter (Fig. 2). No benign or malignant tumors were observed in the perirenal hematoma and extravasated urine. The ureteric calculus and atrophy of the lower half of the left kidney were detected in a follow‐up CT of the parotid carcinoma performed 10 months before (Fig. 3). At this time, there was no urologic consultation regarding ureteric calculus. A self‐discharged urine cytology was negative. The diagnosis was parenchymal rupture of the lower half of the left kidney caused by an obstructive ureteric calculus in an incompletely duplicated renal pelvis and ureter.

**Fig. 4:**
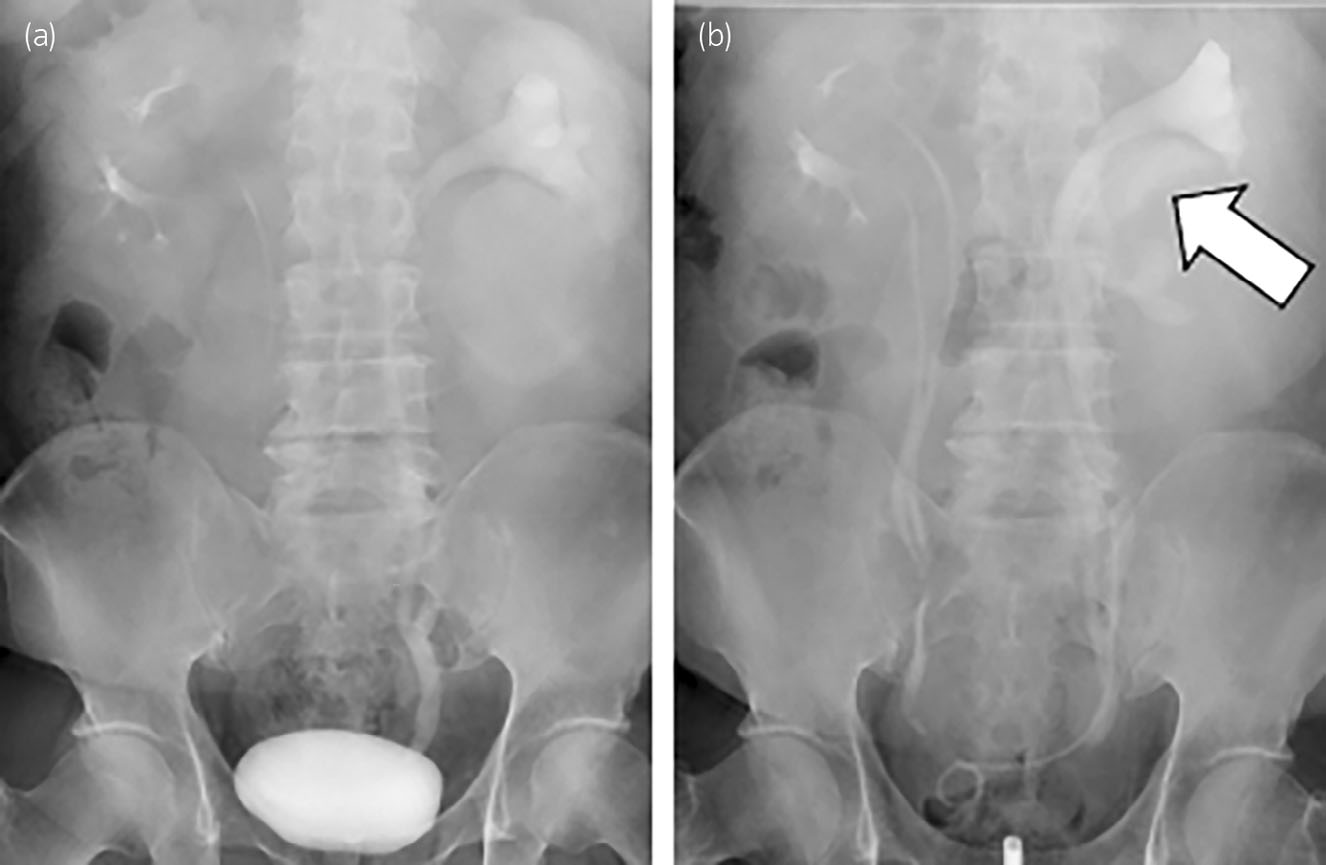
Retrograde pyelography showing bilateral incompletely duplicated renal pelvis and ureter. (a) Before ureteral stenting. (b) After ureteral stenting (white arrow) for lower incompletely duplicated renal pelvis.

At this point, the vital signs of the patient were stable (blood pressure 144/78 mmHg, heart rate 103 beats per min, and body temperature 37°C). The hemoglobin level was 13.1 g/dL (normal range: 13.1–16.3 g/dL), blood urea nitrogen was 19.3 mg/dL (normal range: 8.0–20.0 mg/dL), and creatinine level was 1.17 mg/dL (normal range: 0.65–1.09 mg/dL). We attempted retrograde pyelography and ureteral stenting using a cystoscope with a fluoroscopic guidance under sacral anesthesia. Retrograde pyelography revealed a bilateral incompletely duplicated renal pelvis and ureter. A ureteral stent was placed in the lower part of the renal pelvis to remove the urinary tract obstruction, and to improve pain and mild renal dysfunction (Fig. 4).

The patient was treated with bed rest, hemostatic agents, and empirical antibiotics. The day after admission, his hemoglobin level slightly decreased (10.3 g/dL). However, transfusion was not required because hematuria did not progress, and his vital signs were stable. His left back pain improved over time, and a follow‐up CT on the fifth day of hospitalization did not show an increase in the hematoma. He was discharged 1 week after admission. There were no complications during the follow‐up, and the hematoma had decreased (Fig. S1). After confirming the disappearance of the hematoma, the patient underwent transurethral lithotripsy at another hospital approximately 6 months after discharge from our hospital. The ureteral stent was removed 4 weeks after we performed transurethral lithotripsy.

## Discussion

Although spontaneous parenchymal renal rupture is rare, it is a urologic emergency. Malignancies, tuberculosis, abscesses, calculi, hemophilia, and polycystic kidney disease are causes of parenchymal renal rupture. Abdominal or back pain is a common symptom of parenchymal renal rupture due to retroperitoneal nerve plexus irritation, which causes vomiting in some patients.^11^ Macrohematuria or microhematuria may appear; however, it is no character of spontaneous renal rupture. Its appearance mainly depends on the primary disease, such as ureteric calculi.^12^ In our patient, similar symptoms occurred during transport. The high density of masses in the renal capsule with extravasation into the perirenal space are typical CT findings of spontaneous renal rupture. A CT scan can detect and quantify the extent and location of perirenal hemorrhage. CT finding showed parenchymal renal rupture with a perinephric hematoma in this case.

The upper urinary tract obstruction caused by a distal urinary calculus may have led to increased renal pressure and vulnerability of the renal parenchyma, increasing the risk of parenchymal renal rupture.^13^ In our patient, the calculus was in the ureter in the lower half of the kidney, and the kidneys had already atrophied at least 10 months before. Subsequently, the sudden increase in renal pressure caused by the urinary calculus in the vesicoureteral junction may have resulted in the rupture of the fragile renal parenchyma. Renal calculi can cause parenchymal renal rupture. In addition, renal calculi complicated by infection make the kidney fragile, and it can shatter with multiple calculi.^4^ There have been cases of parenchymal renal rupture in patients following urolithiasis for renal calculi, including extracorporeal shock wave lithotripsy.^1^, ^2^, ^4^

Treatment for spontaneous renal rupture depends on the underlying etiology and the patient's hemodynamic status. In most cases, severe bleeding requires an open surgical intervention. Conservative management without surgical procedure has been described in hemodynamically stable patients with no evidence of persistent bleeding.^2^ Successful renal preservation without nephrectomy was possible because the vital signs were stable, and anemia did not progress. Moreover, the ureteral stent was placed immediately in the lower left half of the kidney, which was the renal rupture site.

The key points in this case were preventing internal renal pressure increases and overflowing urine by implanting a ureteral stent on the affected side. If the renal pelvis pressure had remained elevated, there would have been a risk of bleeding, increased hematuria, urine overflow, and infection, which may have resulted in nephrectomy. In addition, there was no hydronephrosis in the upper half of the left kidney. The reason for the absence of hydronephrosis of the upper half of the left kidney may have been that the ureteric calculus fitted below the confluence of the incomplete duplicated ureter, allowing urine from the upper renal pelvis to flow to the ruptured area. We believe that the absence of hydronephrosis of the upper half of the left kidney was an important factor in the preservation of renal function, which allowed for effective ureteral stenting and conservative treatment in this case. Therefore, although this case is extremely rare, ureteral stenting could be worthwhile if the patient's vital signs are stable. To the best of our knowledge, this is the first case report of conservative treatment with urinary stenting for parenchymal renal rupture due to a ureteric calculus in an incompletely duplicated renal pelvis and ureter. We believe the aggressive treatment of ureteric calculus could be important in such case. The information in this report can contribute further insight into the emergency response.

## Conclusion

In cases of parenchymal renal rupture due to a ureteric calculus in an incompletely duplicated renal pelvis and ureter, placing a ureteral stent for renal preservation is worthwhile.

## Author contributions

Moe Toyoshima: Conceptualization; data curation; writing – original draft; writing – review and editing. Daiki Ikarashi: Conceptualization; data curation; supervision; writing – review and editing. Kie Sekiguchi: Data curation; investigation. Tatsuya Kawamura: Data curation. Arisa Machida: Data curation; investigation. Takeshi Yamaguchi: Data curation; writing – review and editing. Yumeka Arakawa: Data curation. Akito Ito: Data curation. Shigekatsu Maekawa: Data curation; investigation; supervision. Wataru Obara: Supervision; writing – review and editing.

## Conflict of interest

The authors declare no conflict of interest.

## Approval of the research protocol by an Institutional Review Board

Not applicable.

## Informed consent

Written informed consent was obtained from the patient.

## Registry and the Registration No. of the study/trial

Not applicable.

## Supporting information


**Fig. S1** The perinephric hematoma is shown (a) at onset and (b) 1 month after discharge with regression.
