# Supplementary figures and images for: Parenchymal renal rupture due to an obstructive ureteric calculus in an incompletely duplicated renal pelvis and ureter
Source: IJU Case Rep. 2024 Jan 30;7(2):177–80. doi: 10.1002/iju5.12697 (PMC10909126; doi:10.1002/iju5.12697)

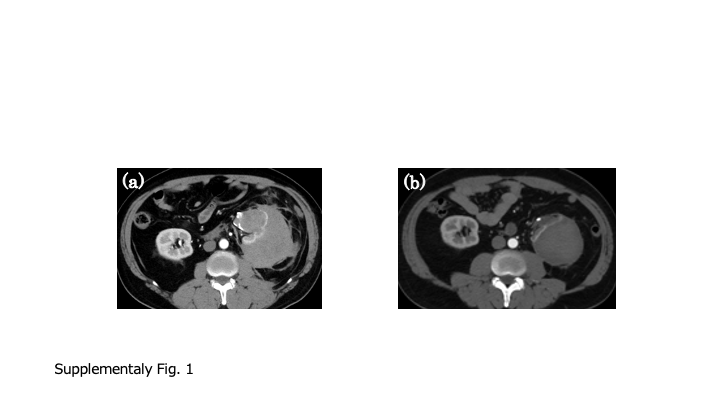

Supplement: Supplementary file 1 — Fig. S1 The perinephric hematoma is shown (a) at onset and (b) 1 month after discharge with regression. [file IJU5-7-177-s001.tiff]
